# *In Vitro* Experimental Assessment of Ethanolic Extract of *Moringa oleifera* Leaves as an *α*-Amylase and *α*-Lipase Inhibitor

**DOI:** 10.1155/2022/4613109

**Published:** 2022-12-29

**Authors:** Adebanke Ogundipe, Babatunde Adetuyi, Franklyn Iheagwam, Keleko Adefoyeke, Joseph Olugbuyiro, Oluseyi Ogunlana, Olubanke Ogunlana

**Affiliations:** ^1^Department of Biochemistry, Covenant University, Canaan Land, P.M.B 1023, Ota, Ogun State, Nigeria; ^2^Coveant Applied Informatics and Communication Africa Centre of Excellence, Covenant University, Canaan Land, P.M.B. 1023, Ota, Ogun State, Nigeria; ^3^Department of Natural Sciences, Biochemistry Unit, Precious Cornerstone University, Ibadan, Oyo State, Nigeria; ^4^Leady Pharma Industries Limited, Sango, Ota, Ogun State, Nigeria; ^5^Department of Industrial Chemistry, Covenant University, Canaan Land, P.M.B. 1023, Ota, Ogun State, Nigeria; ^6^Department of Biological Sciences, Crawford University, Igbesa, Ogun State, Nigeria

## Abstract

**Methods:**

Phytochemical screening, antioxidant activity, *α*-amylase, and *α*-lipase inhibitory assessment were carried out on *Moringa oleifera* extract.

**Results:**

The result of the phytochemical screening revealed the presence of total phenolic, flavonoid, tannin, and alkaloid contents of values 0.070 ± 0.005 mg gallic acid equivalent/g, 0.180 ± 0.020 mg rutin equivalent/g, 0.042 ± 0.001 mg tannic equivalent/g, and 12.17 ± 0.001%, respectively, while the total protein analysis was 0.475 ± 0.001 mg bovine serum albumin equivalent/g. Ferric reducing antioxidant power (FRAP) and total antioxidant capacity (TAC) values were 0.534 ± 0.001 mg gallic acid equivalent/g and 0.022 ± 0.00008 mg rutin equivalent/g, respectively. Diphenyl-2-picrylhydrazyl (DPPH), ABTS (2,2′-azino-bis (ethylbenzothiazoline-6-sulfonic acid)), and nitric oxide (NO) assays showed the extract to have a strong free radical scavenging activity. The 50% inhibitory concentration (IC_50_) values of the lipase and amylase activities of the extract are 1.0877 mg/mL and 0.1802 mg/mL, respectively.

**Conclusion:**

However, *α*-lipase and *α*-amylase inhibiting activity of *M. oleifera* could be related to the phytochemicals in the extract. This research validates the ethnobotanical use of *M. oleifera* leaves as an effective plant-based therapeutic agent for diabetes and obesity.

## 1. Introduction

Diabetes mellitus is a disorder that occurs when there is little or not enough insulin production from the pancreas. It is often regarded as having excessive blood glucose in the blood (hyperglycaemia). According to the International Diabetes Federation, a total of 387 million people were diagnosed with diabetes worldwide in 2014, with the figure expected to rise to 592 million by 2035 [1]. The core remedy for managing diabetes is to lower hyperglycaemia and reduce intestinal glucose absorption through the inhibition of carbohydrate metabolizing enzymes (e.g., *α*-amylase) [2]. The toxicity levels and the high cost of pharmaceutical drugs designed for carbohydrate metabolizing enzyme inhibition have been a major concern [3].

Obesity, a metabolic disorder, occurs as a result of excessive accumulation of fat [4–6]. It is also regarded as an imbalance in energy intake and expenditure [7, 8]. Further complications of obesity can result in type 2 diabetes. Pancreatic *α*-lipase expression is vital in developing obesity towards an advanced state [9]. Phytochemicals such as quercetin, flavonoids, and polyphenols are known to inhibit pancreatic lipase. There is a need to adopt the use of herbs because they are readily available, minimize the side effects caused by synthetic drugs such as orlistat, and also reduce the cost of drug purchases.

Alternative medicines have been adopted for healing several ailments, whereas pharmacological treatment has provided no sustained weight loss with few or no side effects [10, 11]. Therefore, a cost-effective management approach is needed to be adopted to ensure minimal side effects in diabetes and obesity treatment. The therapeutic importance of medicinal plants has accelerated researchers' interest in the discovery of the possible activities of plants to prevent and protect against chronic diseases [11]. Phenols, a bioactive compound found in plants were reported to inhibit *α*-amylase and *α*-lipase; thus, providing it an excellent approach for type 2 diabetes and obesity management [12–16]. Phenolic compounds act by scavenging reactive oxygen created during metabolic reactions in humans. Thus, they are known to fight against cancer, obesity, and diabetes [17]. However, various prospective studies have shown that some compounds such as catechin, isocatechin, flavonoids, flavones, isoflavone, and anthocyanin, have exhibit antiobesity and antidiabetic properties when comparied to *β*-carotene, vitamin E, and vitamin E [18] to have exhibit antiobesity and antidiabetic properties.

*α*-amylase (*α*-1-4-glucan 4-glucanohydrolase, EC 3.2.1.1) is involved in the catalysis of the breakdown of *α*-(4)-D-glycosidic linkage of starch and, therefore, in reducing blood glucose levels. *α*-Lipase (glycerol ester hydrolase EC 3.1.1.3) help to catalyse the breakdown of fats. The digestion, transportation, and processing of dietary lipids are mainly performed by fats. Therefore, diabetes and obesity treatments are targeted towards inhibiting *α*-amylase and lipase enzymes [19].

*Moringa oleifera*, an Indian tree grown in various areas of Mexico, belongs to the *Moringaceae* family, ranging between 5–10 m high. The flowers, seeds, pods, and leaves of the *Moringa* tree have several medicinal benefits used for therapeutic purposes. For instance, the flowers have been studied to have anti-inflammatory activities, the seeds exhibit antihypertensive and liver-protective properties, and the leaves reported to have antimicrobial and hypoglycaemic activities [20]. It comprises three phytocompounds: rutin, quercetin-3-glycoside, and kaempferol, which have been evaluated as a possible mechanism of glucose concentration reduction after ingestion [21–23]. The leaves are mostly taken for self-medication by diabetic and hypertensive patients [24, 25]. The leaves of this plant are mostly used because of their medicinal characteristics, alongside with their hypocholesterolemic and hypoglycemic effects [26, 27]. *M. oleifera* leaves are also rich in ascorbic acid and aid in insulin secretion [22]. Reports of *in vitro* and *in vivo* studies of different extracts of *M. oleifera* have shown them to have antidiabetic and antiobesity properties, but a detailed explanation relating the phytochemicals present in the ethanolic extract of *M. oleifera* to its carbohydrate and lipid metabolizing enzyme inhibiting ability is yet to be properly unveiled. Thus, this investigation was aimed at elucidating the mechanism of action and thereby validating the use of *M. oleifera* ethanolic extract for the prevention and treatment of diabetes and obesity.

## 2. Materials and Methods

*M. oleifera* leaves, part of the whole plant, were harvested from specific locations in Covenant University (N6° 39'53.32644, E3° 9'35.9496), Ogun State, Nigeria. The fresh *M. oleifera* leaves were identified by a botanist in the Biological Sciences, at Covenant University. The voucher numbers were deposited at the Forestry Research Institute of Nigeria (FRIN), Ibadan, Oyo State, Nigeria.

### 2.1. Chemicals and Reagent

Salt of high purity and chemicals of analytical grade were purchased from a reliable source. The following were the list of chemicals used: acetonitrile, sodium phosphate buffer, gallic acid, rutin, pancreatic *α*-amylase, starch solution, ascorbic acid, dinitrosalicylic acid, porcine pancreatic lipase, 4-nitrophenylbutyrate, (2, 2-diphenyl-1-picryl-hydrazyl) DPPH reagent, *α*-amylase, p-nitrophenyl-*α*-D-glucopyranoside, (butylated hydroxyanisole) BHA, potassium ferricyanide and ferric chloride, acarbose, bovine serum albumin, etc.

### 2.2. *M. oleifera* Leaves' Extract Preparation

The selected plants' fresh leaves were air-dried, and an electric blender was used to pulverize them to obtain a fine powder. The powdered form of *M. oleifera* (400 g) was steeped in 90% ethanol in the ratio 1 : 5 (v/v) for three days (72 hours) and then filtered. The obtained filtrates were concentrated using a rotatory evaporator at 50°C to obtain the solvent-soluble fractions [27].

### 2.3. Phytochemical Screening Determination

#### 2.3.1. Total Alkaloid Content Determination

A solution of 200 cm^3^ of acetic acid (10%) diluted in ethanol was added to the sample (in powdered form, 2.5 g). The mixture was placed in a water bath to concentrate the extract to a quarter (1/4^th^) of the original volume after 4 hours. Afterwards, it was filtered and concentrated with 15 drops of ammonium hydroxide until the end of the precipitation. The mixture was subjected to filtration, dried, and weighed after it was left to settle for some hours [22]. Experimental analysis was carried out in triplicate (*n* = 3).

(1) $$\begin{array}{c} Percentage\;of\;alkaloid\left(\%\right)=\frac{weight\;of\;residue}{weight\;of\;sample}|100\text{.} \end{array}$$

#### 2.3.2. Total Phenolic Content Determination

Folin–Ciocalteu (FC) reagent (1 : 10 v/v) of 2.5 mL was mixed with 0.5 mL of extract (1 mg/mL). After 5 minutes of incubation at 25°C, sodium carbonate in the amount of 2 mL (7.5%) was added. The mixture was measured at 765 nm after incubation at 40°C for 30 minutes. The standard used was gallic acid (0.02–0.1 mg/mL), and the extract's phenol was calculated as mg of gallic acid equivalent (mg GAE/g extract) using the standard curve [27]. The experiment was carried out in triplicate (*n* = 3).

#### 2.3.3. Total Flavonoid Content Determination

Two millimetres of distilled water (dH_2_O_2_) was added to 0.15 mL of sample (1 mg/mL), and in addition, 0.15 mL of sodium nitrite (5%) was added. The mixture was subjected to incubation at 25°C for 6 minutes. Aluminium chloride (10%) of 0.15 mL was mixed and further incubated for 5 minutes. To make up to 5 mL, a millimetre of NaOH (4%) and 1.2 mL dH_2_O_2_ were added. Afterwards, the absorbance of the mixture was measured at 420 nm. The standard used was rutin (0.02–0.1 mg/mL) [28]. A flavonoid in the extract was observed by the presence of pink colouration. The content of flavonoids was calculated as mg RE/g extract as rutin equivalent using the standard curve. Experimental analysis was carried out in triplicate (*n* = 3).

#### 2.3.4. Total Tannin Content Determination

An aliquot sample of 0.5 mL (1 mg/mL), dH_2_O_2_ of 3.75 mL, and 0.25 mL of FC reagent (1 : 10 v/v) were mixed together. Lastly, 0.5 mL (35% sodium carbonate) was added to the mixture, and the absorbance was measured. Tannic acid within the concentration range of 0.1–0.00625 mg/mL was used as a reference standard. The extract's total tannin content was calculated using the standard curve and reported as tannic acid equivalent mg TAE/g extract [27]. The experimental analysis was carried out in triplicate (*n* = 3).

#### 2.3.5. Total Saponin Content Determination

An extract of 0.05 mL and 0.25 mL of dH_2_O_2_ were mixed together. Thereafter, vanillin reagents of 0.25 mL (800 mg in 10 mL ethanol) and 2.5 mL of sulphuric acid (72%) were added and further incubated at 60°C for 10 minutes. The absorbance was then read at 544 nm after it was cooled. The standard used was diosgenin, and values were calculated as equivalents of diosgenin (mg DE/g extract) derived from a standard curve [29]. Experimental analysis was done in triplicate (*n* = 3).

#### 2.3.6. Total Protein Content Determination

Alkaline copper sulphate (AlKCuSO_4_) of 2 mL was added to varying concentrations of 0.2 mL of sample and then proceeded for 10 minutes of incubation. A volume of 0.2 mL of FC reagent was mixed and further incubated for 30 minutes. The absorbance of the mixture was measured at 660 nm. BSA (bovine serum albumin) was used as a standard with a varying concentration of 0.05–1 mg/mL. The values were calculated using a standard curve and represented as BSA equivalents (mg BSAE/g extract) [30]. The experiment analysis was carried out in triplicate (*n* = 3).

### 2.4. Antioxidant Activity

#### 2.4.1. Diphenyl-2-Picrylhydrazyl (DPPH)

The DPPH assay was carried out following the method of Iheagwam [27]. A standard/sample of varying concentrations of 0.5 mL was mixed together with 0.5 mL of DPPH (0.1 mM). In the dark, the mixture was set for incubation for 30 minutes. The absorption rate was measured at 517 nm against a blank (methanol), and a DPPH solution was used as a control. Ascorbic acid and silymarin were used as standards (0.00125–0.000019) mg/mL. Radical scavenging activity with a higher value is indicated by a lower absorbance value:

(2) $$\begin{array}{c} \%inhibition\;of\;DPPH\;scavenging\;activity=\frac{Ac-As}{Ac}|100000\text{,} \end{array}$$

where Ac and As denote control and plant extract absorbance, respectively.

#### 2.4.2. FRAP Activity (Ferric Reducing Antioxidant Power)

A sample of 1 mL (0.00625–1) mg/mL was mixed together with 1% potassium ferricyanide [K_3_Fe (CN)^6^] in 2.5 mL, and the mixture was set for incubation at 50°C for 20 minutes. The same volume of trichloroacetic acid was added; 2.5 mL was taken from the mixture, and 2.5 mL of dH_2_O_2_ was added. The absorbance of the mixture was read at 700 nm after 0.5 mL was added to the mixture. The result was compared with ascorbic acid, and values were presented as ascorbic acid equivalents (mg AE/g extract), which were calculated using a standard curve [27]. The experimental analysis was carried out in triplicate (*n* = 3).

#### 2.4.3. 2,2-Azino-bis(3-ethylbenzothiazoline)-6-sulfonic Acid (ABTS) Activity

Potassium persulfate (2.45 mmol/L) and 2,2-azino-bis(3-ethylbenzothiazoline)-6-sulfonic acid (ABTS) (7 mmol/L) were mixed to obtain an ABTS radical solution (ABTS^*|*^) in the ratio 1 : 1 and were incubated for 12–16 hours in the dark. One mL of the radical solution was diluted with the required amount of ethanol/methanol (1 : 89 v/v) to be read using the spectrophotometer to obtain 0.700 ± 0.200 values immediately before use at 745 nm. After that, 0.1 mL of various concentrations of extract (0.0375–1) mg/mL were added to 3.9 mL of ABTS solution and then incubated for 6 minutes.

The absorbance of the solution was read at 745 nm against a blank (methanol); ABTS was used as control and butylated hydroxytoluene (0.0375–1.2 mg/mL) as standard [31]:

(3) $$\begin{array}{c} \%inhibition\;of\;ABTS\;scavenging\;activity=\frac{Ac-As}{Ac}|100\text{,} \end{array}$$

where Ac and As denote control and plant extract absorbance, respectively.

#### 2.4.4. Nitric Oxide (NO)

Phosphate buffer saline (pH 7.4) of 0.5 mL and 2 mL of sodium nitroprusside (10 mM) were mixed with varying concentrations of the sample (0.00006–1) mg/mL. The mixture was incubated for 150 minutes at 25°C. After 30 minutes of incubation, 0.5 mL of the mixture was added to 0.5 mL of Griess reagent (% sulphanilamide, % phosphoric acid, and % naphthyl ethylenediamine dichloride) of and further incubated at the same temperature. The absorbance was read at 546 nm against a phosphate buffer and a control of Griess reagent [32]:

(4) $$\begin{array}{c} \%inhibition\;of\;NO\;scavenging\;activity=\frac{Ac-As}{Ac}|100\text{,} \end{array}$$

where Ac and As denote control and plant extract absorbance, respectively.

#### 2.4.5. Total Antioxidant Capacity (TAC)

One (1) mL of phosphomolybdate reagent (4 mM ammonium molybdate, 28 mM sodium phosphate, and 0.6 M sulphuric acid) was added to a 0.1 mL sample. The absorbance of the mixture was measured at 695 nm after the mixture was set to incubate for 1 hour and 30 minutes. Distilled water was replaced with the sample/standard, and the control used was gallic acid. The extract's phenol content was calculated as mg of gallic acid equivalent (mg GAE/g extract) as derived from the standard curve [27]. The experimental analysis was carried out in triplicate (*n* = 3).

### 2.5. Enzyme Inhibition Study

#### 2.5.1. Carbohydrate Inhibitory Assessment

Amylase solution (2 U/*μ*L in phosphate buffer, 6.8) of a volume of 500 *μ*L added to 250 *μ*L of acarbose/sample. Incubation was set at 37°C for 20 minutes. A 250 *μ*L starch solution (phosphate buffer, 1%, *pH* 6.8) was added to the mixture and further incubated for an hour at 37°C. After incubation, 1 mL of dinitrosalicylic acid (DNSA) reagent was added and left to boil for 10 minutes. The mixture was taken at 540 nm against buffer and DNSA as blank [33]:

(5) $$\begin{array}{c} \%inhibition\;of\;alpha\;amylase\;activity=\frac{Ac-As}{Ac}|100\text{,} \end{array}$$

where Ac and As denote control and plant extract absorbance, respectively.

#### 2.5.2. Lipase Inhibitory Assessment

In a 96-well plate, 164 mL of assay buffer and 6 mL of pancreatic lipase solution were mixed. Also, 20 mL of extract/orlistat was pipetted and set for incubation for 10 minutes at 37°C. Thereafter, 10 mL of the substrate was added and set for incubation again at 37°C for 15 minutes. The absorbance of the mixture was read at 405 nm [34]:

(6) $$\begin{array}{c} \%inhibition\;of\;alpha\;lipase=\frac{Ac-As}{Ac}|100\text{,} \end{array}$$

where Ac and As denote control and plant extract absorbance, respectively.

## 3. Results

### 3.1. Voucher Referencing and Extraction Yield of the Selected Plants

The present study was conducted with an aqueous 95% ethanol extract of the selected medicinal leaves. The percentage yield of *M. oleifera* (Mo/Bio/H816) was calculated at 27.78%.

### 3.2. Phytochemical Screening and Total Protein Content of *M. oleifera* Leaf Extract

Leaves extract showed that phytochemicals were present, which are stated in Table 1. These phytochemicals include phenols, flavonoids, tannins, alkaloids, and saponins. *M. oleifera* leaves were reported to have high nutritional value; this study revealed the presence of high protein content (0.475°0.001 mg BSAE/g) in the extract.

### 3.3. Antioxidant Analysis

**Figure 1:**
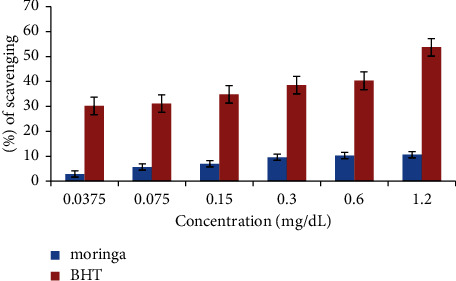
Antioxidant activity of *M. oleifera* on the ABTS radical. SCV (%) means percentage scavenging activity.

To investigate the antioxidant ability of the extract from *M. oleifera* leaves, antioxidant assays such as DPPH, nitric oxide, FRAP, TAC, and ABTS were carried out. The results of FRAP and TAC showed that 0.022°±°0.00008 mg RE/g and 0.534°±°0.001 mg GAE/g, respectively, as shown in Table 2. The inhibitory percentage of ABTS at the highest concentration (1.2 mg/mL) is 10.67%, as shown in Figure 1. Moreover, the 50% inhibitory concentrations (IC_50_) values for DPPH, nitric oxide, and ABTS values of *M. oleifera* shown in Table 3 are 0.0002 mg/mL, 0.6577 mg/mL, and 8.3036 mg/mL, respectively.

### 3.4. Enzyme Inhibitory Assessment

#### 3.4.1. Percentage Inhibitory Effect of Different Concentrations of *M. oleifera* Extract on *α*-Amylase Enzyme Activity

**Figure 2:**
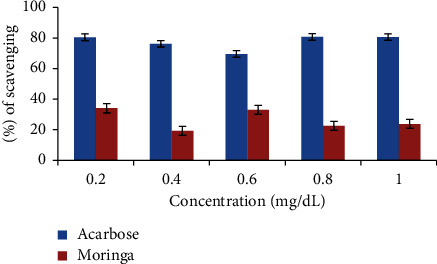
Effect of *M. oleifera leaf* extract on *α*-amylase activity.

*M. oleifera* extract inhibits the activity of the *α*-amylase enzyme in a concentration-dependent manner. The percentage inhibitory values of the extract were evaluated to be 34%, 19%, 33%, 22.58%, and 23.79% at 0.2 mg/mL, 0.4 mg/mL, 0.6 mg/mL, 0.8 mg/mL, and 1 mg/mL concentrations, respectively. The highest percentage inhibitory values of acarbose were estimated at 80.72% at 0.8 mg/mL concentration, as shown in Figure 2. The IC_50_ values of the amylase inhibitory activity of the extract were 0.1802 mg/mL and that of acarbose were 0.1215 mg/mL, as shown in Table 4.

### 3.5. Percentage Inhibitory Effect of Different Concentrations of *M. oleifera* Extract on *α*-Lipase Enzyme Activity

**Figure 3:**
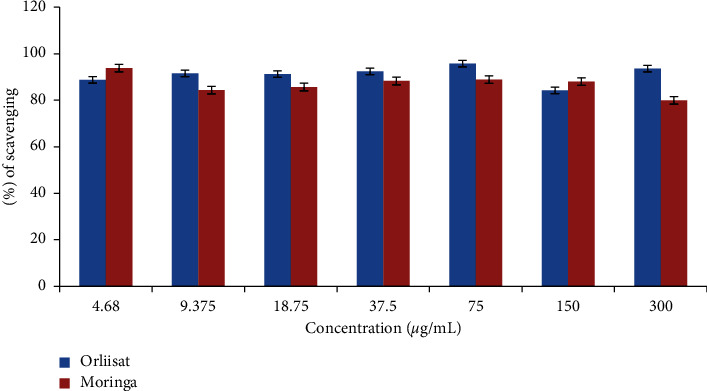
Effect of *M. oleifera leaf* extract on *α*-lipase activity.

*M. oleifera* extracts had a concentration-dependent inhibitory effect on the *α-lipase* enzyme. The highest percentage inhibitory value was 93.84% at 4.68 *μ*g/mL concentration, and the lowest percentage inhibitory value was 79% at a concentration of 300 *μ*g/mL. However, orlistat has the highest percentage inhibitory value of 95.76% at 75 *μ*g/mL concentration, as shown in Figure 3. The IC_50_ values of pancreatic lipase inhibitory activity of the extract were 1.0877 mg/mL, and orlistat (the standard drug) was 0.8170 mg/mL, as shown in Table 4.

## 4. Discussion

*In vitro* studies in all fields of biology are aimed at elucidating the mechanisms by which biological substances perform their roles within a cell [35]. The uses of medicinal plants in delivering cost-effective therapy for a variety of ailments are due to the existence of secondary metabolites [36]. Plants contain bioactive compounds, which have been shown in studies to have a variety of therapeutic properties [37].

*M. oleifera* has proven to have various antidiabetic, antiobesity, antioxidant, and anti-inflammatory effects. It was reported to contain a huge amount of proteins, oils, potassium, calcium, carbohydrates, amino acids, and phenolic compounds (such as rutin, kaempferol, p-coumaric acid, and quercetin). The antidiabetic and antiobesity pharmacological properties of *Moringa oleifera* are a result of its high constituents of flavonoid, glucoside, and glucosinolate [38].

The amphipathic nature of ethanol allows for the breakdown of both polar and nonpolar elements in plants [39]. It was also shown to be the best choice for extracting active principles. The ethanolic extract of *M. oleifera* showed phytochemical constituents such as phenol, flavonoid, saponin, and tannin, which could be related to the presence of high phytochemical content in this study. Furthermore, the onset of diabetes can be delayed, as revealed in experimental studies with *M. oleifera* leaves extract with high phenolic content [40, 41], which is also in agreement with the high phenolic content revealed in this research. The therapeutic potential of phytocompounds in *M. oleifera* has been reported *in vitro* and *in vivo* studies for reducing the risk of chronic diseases [42].

Natural antioxidants, which can be found in a variety of foods and medicinal plants such as fruits, vegetables, beverages, herbs, and spices, play a major role in the human diet, particularly in preventing cellular damage (oxidative stress) [43]. Tannins and saponins have been discovered to have strong antioxidant properties [44], which could be responsible for the radical scavenging ability of *M. oleifera* ethanolic extract to scavenge nitric oxide, diphenyl-2-picrylhydrazyl (DPPH), and 2,2-azino-bis(3-ethylbenzotiazoline)-6-sulfonic acid (ABTS) activity radicals in this present study. Moreover, the phytochemicals present in *M. oleifera* leaves have been proven to have antioxidant phytochemicals present in them.

Alpha amylase and pancreatic lipase inhibition are one therapeutic method to reduce hyperglycaemia and obesity [45]. *In vivo* investigation, *M. oleifera* was proven to have antihyperglycaemic properties via the release of insulin such as sulfonylureas and meglitinides to inhibit ATP-sensitive potassium channels in the residual beta cell channels [46]. High inhibitory effects were shown on *α-*amylase by *M. oleifera* more than the reference drug (acarbose) as a result of the saponin, phenolic, and flavonoid content. This is related to the result of the *α-*amylase inhibitory assessment revealed in this study.

*M. oleifera* ethanolic leaf extract effectively inhibited pancreatic lipase using orlistat as the standard. *In vitro*, *in vivo,* and clinical studies have shown that *M. oleifera* has antiobesity properties. Bioactive compounds such as polyphenols and quercetin have antiobesity activities and are great pancreatic lipase inhibitors [47]. This result validates the pancreatic lipase inhibitory property of *M. oleifera* ethanolic leaves.

## 5. Conclusion

*M. oleifera* leaf extract has been validated to be an excellent *α-*amylase and *α-*lipase inhibitor with properties suitable for the prevention and treatment of diabetes mellitus and obesity when consumed. The bioactive compounds such as flavonoid, saponin, tannin, etc. present in *M. oleifera* ethanolic leaves extract as reported in this present and other previous studies were shown to possess antioxidant, antidiabetic, and antiobesity activity, which could be responsible for its potent radical scavenging ability and effective inhibition of *α*-amylase and pancreatic lipase. These natural bioactive compounds and *M. oleifera* powdered leaves can likewise be included in foods and used as drugs for weight loss management. Moreover, a natural deep eutectic solvent may be adopted in extract preparation for better extraction, low toxicity, and probably to achieve unique chemical compounds. Clinical trial studies can be explored further to maximise the gains in the use of *M. oleifera* leaves in humans.

## Figures and Tables

**Table 1:** Phytochemicals screening and total protein content of *M. oleifera*.

Phytochemicals and protein content	*M. oleifera*
TPC (mg GAE/g)	0.070 ± 0.005
TFC (mg RE/g)	0.180 ± 0.020
TSC (mg DE/g)	1.929 ± 0.193
TTC (mg TAE/g)	0.042 ± 0.001
TAC (%)	12.17 ± 0.001
TPC (mg BSAE/g)	0.475 ± 0.001

Means and SEM (standard error of the mean) are used to express the values. GAE, RE, DE, TAE, BSAE, TPC, TFC, TSC, TTC, and TAC represent gallic acid equivalent, rutin equivalent, diosgenin equivalents, tannic acid equivalents, bovine serum albumin equivalents, total phenolic content, total flavonoid content, total saponin content, total tannin content, and total alkaloid content, respectively.

**Table 2:** Antioxidant activity of *M. oleifera*.

Antioxidant assays	*M. oleifera*
FRAP (mg RE/g)	0.022 ± 0.00008
TAC (mg GAE/g)	0.534 ± 0.001

TAC = total antioxidant capacity; FRAP = ferric reducing antioxidant power; RE = rutin equivalent; GAE = gallic acid equivalent.

**Table 3:** DPPH and nitric oxide IC_50_ values of *M. oleifera*.

	DPPH (mg/mL)	Nitric oxide (mg/mL)	ABTS (mg/mL)
Ascorbic acid	0.0003	0.0198	—
*M. oleifera*	0.0002	0.6577	8.3036
BHT	—	—	1.0181

**Table 4:** Amylase and lipase IC_50_ values of *M. oleifera*.

	Lipase	Amylase
Acarbose	—	0.1215
ORLISTAT	0.8170	—
*M. oleifera*	1.0877	0.1802

## Data Availability

All the data supporting this study are available upon request.
